# Functional evidence of Hv1-mediated proton flux in cardiac mitochondria

**DOI:** 10.1080/19336950.2026.2728231

**Published:** 2026-09-14

**Authors:** Rayen De Fazio, Clara Ventura, Paulina Finochietto, Ernesto A. Aiello, Carolina Jaquenod De Giusti, Verónica Milesi

**Affiliations:** a Centro de Investigaciones Cardiovasculares ‘Dr. Horacio E. Cingolani’ CCT-La Plata/CONICET, Facultad de Ciencias Médicas, UNLP, La Plata, Argentina; b Departamento de Ciencias Biológicas, Instituto de Estudios Inmunológicos y Fisiopatológicos (IIFP), UNLP, CONICET, asociado CIC PBA, Facultad de Ciencias Exactas, La Plata, Argentina

**Keywords:** Hv1 channel, mitochondria, pH, membrane potential, heart

## Abstract

Mitochondria are essential for cardiac myocyte function, providing the continuous ATP supply required for contraction and cellular homeostasis. Regulation of the proton motive force, which is critical for ATP synthesis, depends on multiple ion transport mechanisms, including those mediating H^+^ flux. The voltage-gated proton channel (Hv1), encoded by the *Hvcn1* gene, is known to regulate cytosolic pH and membrane potential in several cell types. Here, using isolated mouse cardiac mitochondria, we demonstrate for the first time that Hv1 is functionally expressed in cardiac mitochondria. Pharmacological inhibition of Hv1 enhanced matrix alkalinization and induced mitochondrial hyperpolarization during succinate-driven mitochondrial energization. Importantly, a similar hyperpolarization was also observed under basal conditions in intact cardiac myocytes.

## Introduction

Mitochondria play a fundamental role in cardiac myocytes, as the heart relies heavily on a continuous supply of ATP to sustain contractile function and cellular homeostasis. These organelles occupy nearly 30% of the cell volume and are tightly regulated to ensure efficient oxidative phosphorylation [1]. In this process, electrons derived from oxidizable substrates such as glucose and fatty acids are transferred through a series of electron carriers in the respiratory chain, ultimately reducing molecular oxygen to water. The energy released during this transfer drives proton-pumping activities that establish a proton motive force across the inner mitochondrial membrane, which is essential for mitochondrial ATP synthesis. This electrochemical gradient – comprising both electrical and proton (H^+^) concentration gradients – is finely regulated by various structures that mediate H^+^ flux through mitochondrial membranes, along with other ion fluxes that also influence the membrane potential [2].

The voltage-gated proton channel (Hv1), encoded by the *Hvcn1* gene and expressed in the plasma membrane, plays a relevant role in cytosolic pH and membrane potential regulation across various cell types [3]. Its function is primarily associated with NADPH oxidase activity and has been extensively documented in phagocytic immune cells [4]. More recently, two independent research groups have reported the presence of Hv1 in mitochondria: Patel *et al*. [5] identified Hv1 in mitochondria from tubular renal cells, while Coe *et al.* [6] provided evidence of its expression in mitochondria from CD8^+^ T cells. The functional implication of mitochondrial Hv1 in renal cells is related to NAD(P)H oxidase regulation in response to intracellular Na^+^ levels, whereas in naive CD8+ T cells the channel is involved in maintaining mitochondrial fitness during the transition to activated T cells. This novel localization of Hv1 and its associated functions suggests that this channel may participate in the regulation of mitochondrial bioenergetics and membrane potential in physiological and pathological contexts.

Here, using isolated mouse cardiac mitochondria and measurements of mitochondrial membrane potential in intact cells, we demonstrate for the first time that Hv1 is functionally expressed in cardiac mitochondria and contributes physiologically to mitochondrial pH and membrane potential regulation. Our findings support a role for Hv1-mediated proton flux both during succinate-driven mitochondrial energization and under basal conditions.

## Materials and methods

### Mitochondria isolation

Twelve-week-old male C57BL/6J mice were anesthetized with isoflurane and sacrificed by cervical dislocation. Following euthanasia, the hearts were excised and ventricular mitochondria were isolated by differential centrifugation as previously described [7]. Briefly, ventricles were homogenized in ice-cold STE1 buffer (250 mM sucrose, 5 mM Tris/HCl, 2 mM EGTA, 0.1% bovine serum albumin (BSA), pH 7.4), and mitochondria were isolated through a series of centrifugation steps. The resulting mitochondrial pellet was resuspended in Baines buffer (20 mM MOPS, 120 mM KCl, 5 mM KH_2_PO_4_, pH 7.4) for downstream analyses.

### Isolation of cardiac myocytes from adult mouse heart

Mouse ventricular adult myocytes were isolated as previously described [8]. Briefly, 12-week-old C57BL/6J mice were anesthetized under 4% isoflurane, and the chest cavity was opened to expose the heart. The descending aorta was cut, and the heart was immediately flushed by injection of EDTA buffer (NaCl 130 mM, KCl 5 mM, NaH_2_PO_4_ 0.5 mM, HEPES 10 mM, glucose 10 mM, BDM 10 mM, taurine 10 mM, EDTA 5 mM, pH 7.8) into the right ventricle. The ascending aorta was clamped, and the heart was transferred to a 60-mm dish containing fresh EDTA buffer. Digestion was achieved by sequential injection of EDTA buffer, perfusion buffer (NaCl 130 mM, KCl 5 mM, NaH_2_PO_4_ 0.5 mM, HEPES 10 mM, glucose 10 mM, BDM 10 mM, taurine 10 mM, MgCl_2_ 1 mM, pH 7.8), and digestion buffer (collagenase type II 0.5 mg/ml, protease type XIV 0.05 mg/ml in perfusion buffer) into the left ventricle (LV). Ventricles were then separated, and cellular dissociation was completed by gentle trituration. Enzyme activity was inhibited by the addition of stop buffer (FBS 5% v/v in perfusion buffer). The cell suspension underwent 4 sequential rounds of gravity settling using 3 intermediate calcium reintroduction buffers. Cells were subsequently transferred from perfusion buffer to Tyrode buffer (NaCl 140 mM, KCl 5 mM, MgCl_2_ 1 mM, glucose 10 mM, HEPES 10 mM, pH 7.4) containing 1 mM Ca^2+^.

All experiments were performed in accordance with the Guide for Care and Use of Laboratory Animals and approved by the Ethics Committee of the Faculty of Medicine, La Plata, Argentina Institutional Animal Care and Use Committee (CICUAL P07-01–2023).

### Hv1 expression in isolated mitochondria

#### Spectral flow cytometry

Equal amounts of isolated mitochondria were transferred into separate tubes and incubated for 30 min at 37°C with 600 nM MitoTracker Red CMXRos (Invitrogen, M7512) in Baines buffer. After incubation, samples were centrifuged at 10,000 ×g for 10 min at 4°C, and washed with Baines buffer. Isolated mitochondria were resuspended in 4% paraformaldehyde (PFA) dissolved in Baines buffer and incubated for 15 min at room temperature. Samples were washed twice with Baines buffer and incubated with a blocking and permeabilization solution containing 0.1% Triton X-100 and 5% BSA in Baines buffer for 30 min at room temperature. Following an additional wash, mitochondria were incubated overnight at 4°C with the anti-Hv1 primary antibody (Invitrogen, PA5-24964, 1:30) diluted in Baines buffer containing 0.1% Triton X-100 and 1% BSA. After two washes, samples were incubated for 60 min at room temperature with goat anti-Rabbit IgG (H+L) secondary antibody conjugated to Alexa Fluor 488 (Invitrogen, A11008; 1:200) prepared in the same buffer. Finally, mitochondria were washed and resuspended in 150 µl of Baines buffer. Samples were analyzed using a Northern Lights (Cytek) spectral flow cytometer. All steps were performed on ice, and all solutions were filtered through 0.2 µm filters. Reference controls included unstained (only secondary antibody) and single-dye controls. Laser calibration was performed with Cytek SpectroFlo® QC Beads (Cytek, B7-10001). Sensitivity and size-resolution ranges were established using ApogeeMix beads (ApogeeFlow, 1527). Mitochondria were identified and gated based on size and MitoTracker Red CMXRos fluorescence relative to unstained controls.

#### Immunoblot

Isolated mitochondrial preparations were suspended in a RIPA buffer supplemented with protease and phosphatase inhibitor cocktails (Roche). Total protein concentration was determined using the Bradford assay. Equal amounts of protein were separated by electrophoresis on a 10% sodium dodecyl sulfate-polyacrylamide gel (SDS-PAGE) and transferred to PVDF membranes. Membranes were then blocked with 5% nonfat dry milk in TBS-T (Tris-buffered saline, 0.1% Tween-20) for 1 h at room temperature, then incubated overnight at 4°C with the following primary antibodies: anti-VDAC (Invitrogen, PA1-954A, 1:500) and anti-Hv1 (Invitrogen, PA5-24964 1:500). After washing, membranes were incubated with horseradish peroxidase (HRP)-linked anti-rabbit IgG (Santa Cruz Biotechnology, sc-2317, 1:5000) of anti-mouse IgG (Thermo Fisher, 62–6520, 1:4000) for 1 h at room temperature. Protein bands were visualized with Chemiluminescent HRP Substrate (Millipore) using a ChemiDoc Image Station (Bio-Rad).

### Measurement of intramitochondrial pH determination in isolated mitochondria

To assess intramitochondrial pH, 50 µg of isolated mitochondria were incubated with BCECF-AM 15 µM (Invitrogen, B1170), a ratiometric pH-sensitive fluorescent dye, for 20 min at room temperature [9]. Following incubation, samples were centrifuged at 8,000 ×g for 10 min, and the pellet was resuspended in a buffer solution containing 100 mM KCl, 10 mM TES, 30 µM EGTA, 10 µM rotenone at pH 7.2.

Mitochondria aliquots were subsequently treated with either 10 μM of 5-chloro-2-guanidinobenzimidazole (ClGBI), a widely used inhibitor of the Hv1 channel [10], or the corresponding volume of DMSO (vehicle control), for 10 min at 30°C. Using the same protocol, a separate set of mitochondria was incubated with 10 µM HOE-642, a selective inhibitor of the Na^+^/H^+^ exchanger isoform 1 (NHE1) [11] or the corresponding volume of DMSO (vehicle control). BCECF fluorescence was recorded at an emission wavelength of 535 nm using dual excitation wavelengths of 440 nm and 485 nm at 30°C with continuous stirring using a Varioskan Multimode Microplate Reader (ThermoFisher). After 10 min of incubation and 1 min of basal fluorescence measurement (Fi), 5 mM of succinate was added, and fluorescence was measured for an additional 3 min with measurements taken every 10 s. Intramitochondrial pH was estimated from the ratio of fluorescence intensities obtained with excitation at 485 and 440 nm and emission at 535 nm.

### Measurement of mitochondrial membrane potential in isolated mitochondria

Mitochondrial membrane potential (ΔΨm) was evaluated by measuring rhodamine-123 (Rh-123; Sigma-Aldrich, R8004) fluorescence. Rh-123 was used at a final concentration of 100 nM. The dye was excited at 503 nm, and fluorescence emission was recorded at 527 nm. Fluorescence intensity was monitored in a continuously stirred assay medium (Baines’s buffer, 100 nM Rh-123, 5 mM succinate, and 5 µM rotenone) before and after the addition of 50 µg of isolated mitochondria preincubated for 10 min with either DMSO (vehicle), 10 µM ClGBI, or 5 µM CCCP, which was used as a positive control to induce mitochondrial membrane depolarization. Under these conditions, mitochondrial energization promotes Rh-123 accumulation and fluorescence quenching, as originally described by Emaus et al. [12] and further characterized in isolated mitochondria by Baracca et al. [13]. Accordingly, a decrease in fluorescence reflects Rh-123 accumulation by polarized mitochondria. Relative to the control condition, a greater decrease in fluorescence indicates mitochondrial hyperpolarization, whereas a smaller decrease indicates mitochondrial depolarization, as observed in the presence of CCCP and previously described by Baracca et al. [13]. Fluorescence (F) was recorded using a Varioskan Multimode Microplate Reader and results were expressed as the F/Fi ratio, where Fi corresponds to the last fluorescence value recorded prior to the addition of mitochondria.

### Measurement of mitochondrial membrane potential in intact myocytes

In isolated cardiomyocytes, mitochondrial membrane potential (ΔΨm) was assessed using tetramethylrhodamine methyl ester (TMRM). Cardiomyocytes were loaded with 50 nM TMRM for 10 min at room temperature and subsequently maintained in the perfusion solution containing 10 nM TMRM throughout the experiments. As a lipophilic cationic probe, TMRM accumulates within mitochondria in a ΔΨm-dependent manner. Under non-quenching conditions, changes in TMRM fluorescence reflect changes in mitochondrial accumulation of the probe; therefore, an increase in fluorescence is consistent with mitochondrial membrane hyperpolarization, whereas a decrease is consistent with depolarization. Some cells were also loaded with 1 µM DiBAC [4] 3 to monitor changes in plasma membrane potential during the experiments.

Cells were initially superfused for 5 min with Tyrode buffer containing DMSO, and baseline image acquisition was performed for 1 min. Cardiomyocytes were then perfused with Tyrode buffer containing either DMSO or ClGBI (10 μM), and additional image acquisitions were performed after 5 min and 10 min of treatment for 2 min and 1 min, respectively. At the end of the protocol, CCCP 10 μM was added, and after 2 min an additional 1 min acquisition was performed.

Confocal imaging was performed using a Leica Stellaris 8 microscope equipped with a 63x objective. Images were acquired at 1024 × 1024-pixel resolution with a scanning speed of 200 Hz. TMRM fluorescence was excited at 570 nm and detected at 584 nm. Time-series acquisition was performed at a sampling frequency of 1 frame every 10.2 s.

Image analysis was performed using ImageJ. Mean TMRM fluorescence intensity (mean gray value) was quantified within a region of interest encompassing the entire cardiomyocyte and normalized to the initial fluorescence recorded in the same cell.

### Statistical analysis

Data are presented as mean ± SEM. Time-course data were analyzed using a two-way repeated-measures ANOVA, with time and treatment as fixed factors and subject (mouse or myocyte) treated as the repeated-measures factor (Graph Prism 8). When the assumption of sphericity was violated, statistical significance was determined using the Geisser–Greenhouse correction. Statistical significance was set at *p < 0.05*.

## Results

**Figure 1.**
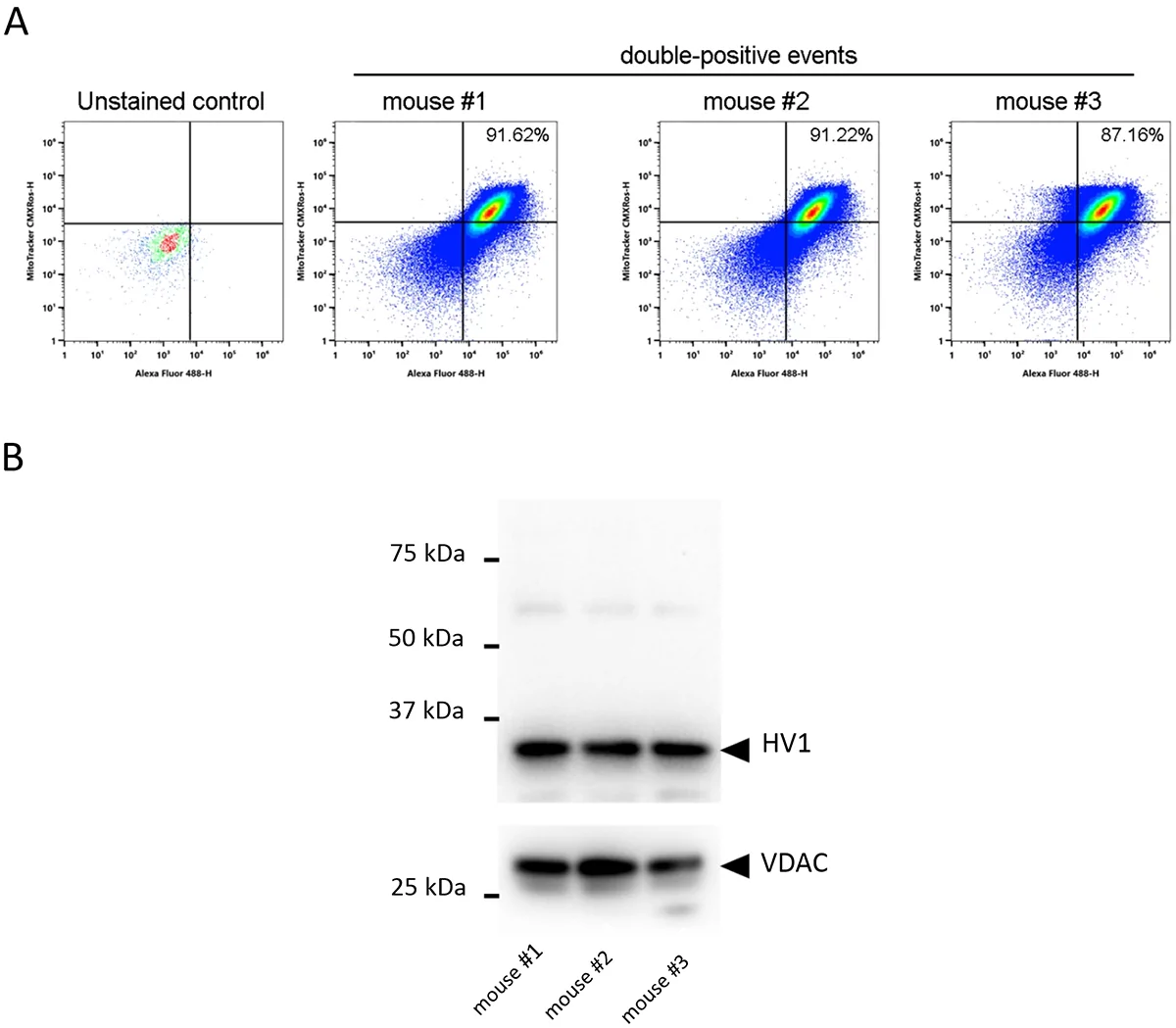
A: MitoTracker and Alexa 488 fluorescence (Hv1) detected in size-defined mitochondrial population (P1). Representative unstained control (only secondary antibody), and double-positive events detected in mitochondrial preparations (*n* = 3). The percentage of MitoTracker+ events expressing the Hv1 channel is indicated. B: expression of the Hv1 channel in isolated mitochondria preparations (*n* = 3) determined by western blot. VDAC determination was used as a mitochondrial marker.

To investigate the presence and function of the Hv1 channel in cardiac mitochondria, we performed experiments on isolated mitochondrial preparations from mouse ventricular myocytes. The samples were labeled with MitoTracker Red CMXRos, a fluorescent dye that selectively accumulates in these organelles and in parallel, with an antibody directed against the Hv1 channel used to detect its expression. Fluorescence signals were analyzed using spectral flow cytometry. Importantly, because the experiments were performed on isolated mitochondrial preparations, contamination from intact cells and other cellular components is expected to be negligible. Under these conditions, spectral flow cytometry enables the identification of mitochondrial events based on a combination of size and fluorescence criteria. The size-resolution range of the instrument was first established using ApogeeMix beads (Figure S1A). Buffer-only controls showed events around 180 nm (Figure S1B) while the events in mitochondrial preparations displayed a size distribution around 1300 nm, consistent with the expected mitochondrial diameter (Figure S1C). The size-defined population (P1) was selected and further validated by positive MitoTracker labeling, ensuring mitochondrial identity. Figure 1(A) shows representative dot plots from unstained control and three independent experiments (*n* = 3 mice). Events positive for both MitoTracker Red and Hv1 (MitoTracker Red^+^/Hv1^+^) indicate mitochondria expressing the Hv1 protein.

Moreover, immunoblot analysis was performed on isolated mitochondrial fractions using an anti-Hv1 antibody. A band at around 30 kDa, consistent with the expected molecular weight of Hv1, was detected in all mitochondrial preparations where VDAC was used as a mitochondrial marker. Figure 1(B) shows representative blots from three different mitochondrial isolations (*n* = 3 mice). The purity of mitochondrial preparations was evaluated using an anti-NaKATPase antibody (Figure S2A). As a positive control for Hv1 expression, total lysates from Jurkat T cells were analyzed showing a similar Hv1-immunoreactive band at 30 kDa along with additional higher molecular weight bands that may correspond to dimeric forms of the channel (Figure S2B).

**Figure 2.**
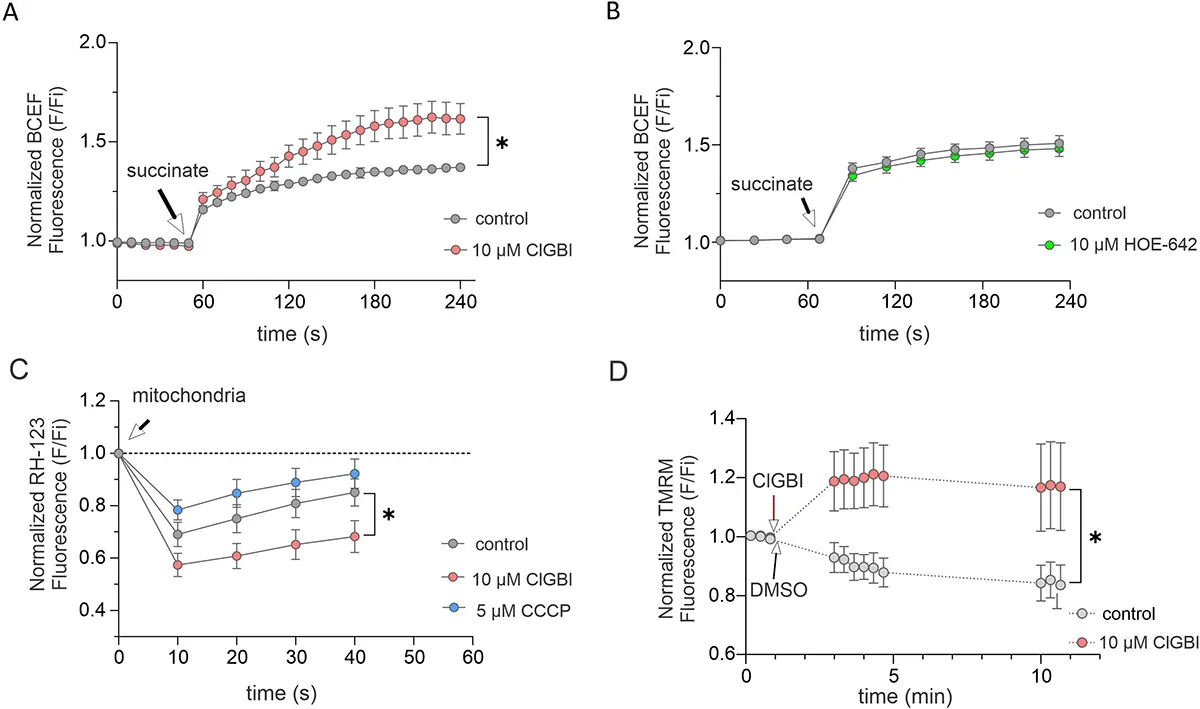
Normalized mean ± SEM of BCECF fluorescence ratio (485/440 nm) values in control conditions (DMSO) and in presence of 10 µM ClGBI (A) or HOE-642 (B). Data are normalized to the initial value of BCECF fluorescence ratio (F/fi), *n* = 5 mice each performed in triplicate. C: normalized mean ± SEM of Rh-123 fluorescence in control conditions (DMSO), with 10 µM ClGBI and in presence of 5 µM CCCP. Data are normalized to the last value prior mitochondrial addition (F/fi), *n* = 8 mice, each performed in triplicate. D: mean ± SEM of TMRM fluorescence in control conditions and after perfusion of myocytes with DMSO (*n* = 8) or 10 µM ClGBI (*n* = 13) isolated from two mice. Fluorescence values correspond to the mean fluorescence intensity (mean gray value) measured for each cardiomyocyte and are expressed relative to the initial fluorescence recorded in the same cell. In all cases, * indicates statistical significance at *p* < 0.05, analyzed using a two-way repeated-measures ANOVA with time and treatment as fixed factors and subject (mouse or myocytes) treated as the repeated-measures factor.

Then, based on the key biophysical properties of the Hv1 channel – namely, its proton permeability and voltage sensitivity – we examined whether this channel contributes to the regulation of mitochondrial pH and mitochondrial membrane potential. To assess its role in pH regulation, mitochondria preloaded with the BCECF-AM probe were energized using a validated protocol that stimulates the electron transport chain (ETC) with succinate [9]. As shown in Figure 2(A), the addition of succinate induced a rapid alkalinization of the mitochondrial matrix, indicated by an increase in the BCECF fluorescence ratio that reached a steady state after approximately 3 min (control condition). When the same protocol was applied in the presence of 10 µM ClGBI, a known Hv1 inhibitor, the alkalinization was significantly greater. Time-course data were analyzed using a two-way repeated-measures ANOVA, revealing a significant effect of time, ClGBI treatment, and time × treatment interaction.

To further examine whether other proton-handling mechanisms could account for a similar effect, we applied the same protocol in the presence of HOE-642, a selective inhibitor of the Na^+^/H^+^ exchanger (NHE1), which has also been reported to localize to mitochondrial membranes [14]. Inhibition of NHE1 did not affect either the time course or the magnitude of the pH changes following succinate addition (Figure 2(B)).

We next investigated whether Hv1-mediated H^+^ flux influences mitochondrial membrane potential (ΔΨm) during succinate-driven respiration. ΔΨm was assessed by monitoring Rh-123 fluorescence, a cationic dye that accumulates within the mitochondrial matrix in a ΔΨm-dependent manner. At higher membrane potentials, increased dye accumulation leads to self-quenching and a decrease in fluorescence intensity. Therefore, reductions in fluorescence (expressed as F/Fi) are indicative of mitochondrial hyperpolarization.

As shown in Figure 2(C), the addition of mitochondria to a medium containing Rh-123 and succinate induced a rapid decrease in Rh-123 fluorescence. As it is possible to observe, in presence of 10 µM ClGBI, the fluorescence decreased further compared to control conditions (DMSO), consistent with a more polarized mitochondrial state. As a positive control, 5 µM CCCP induced the expected membrane depolarization. Time-course of mean ± SEM data in control condition and in presence of ClGBI were analyzed using a two-way repeated-measures ANOVA. This analysis revealed a significant effect of time, ClGBI treatment, and time × treatment interaction, indicating that the temporal profile of the response differed between conditions (Figure 2(C)).

To determine whether the effect of Hv1 inhibition on mitochondrial membrane potential could also be detected in a more physiological context, we evaluated ΔΨm in intact cells in basal conditions using TMRM in the non-quenching mode. Under these conditions, an increase in TMRM fluorescence reflects increased mitochondrial accumulation of the probe and is consistent with mitochondrial hyperpolarization, while a fluorescence decrease indicate depolarization. Accordingly with the results obtained in isolated mitochondria, ClGBI treatment induced a significant increase in TMRM fluorescence, consistent with mitochondrial hyperpolarization, compared with vehicle-treated cells (Figure 2(D) and Figure S3). Control experiments showed that ClGBI had no significant effect on plasma membrane potential compared to the vehicle, measured by DiBAC_4_ [3] fluorescence intensity. After 10 min of incubation, the final values of DiBaC_4_ [3] fluorescence intensity normalized to the initial one were 0.83 ± 0.31 in control conditions and 0.65 ± 0.11 in presence of 10 µM ClGBI (*n* = 4–5 *p* > 0.05; Student t test).

## Discussion

In this work, the combined results from flow cytometry, immunoblotting, and functional studies in isolated cardiac mitochondria and intact myocytes provide converging evidence supporting the existence of an Hv1-mediated proton conductance in cardiac mitochondria. Our functional data are consistent with an Hv1-mediated proton influx contributing to matrix pH homeostasis during succinate-driven mitochondrial energization. We also found that NHE1, another proton-handling mechanism that could participate in mitochondrial pH regulation, does not contribute to the succinate-induced matrix pH response. Furthermore, the consistent findings obtained in isolated mitochondria and under basal conditions in intact myocytes indicate that Hv1-mediated proton influx contributes to the regulation of ΔΨm. The hyperpolarization observed following Hv1 inhibition is consistent with previous reports showing that HVCN1-deficient CD8^+^ T cells exhibit a higher mitochondrial membrane potential than WT naïve CD8^+^ T cells [6]. Collectively, these findings suggest that Hv1 contributes to intramitochondrial pH regulation and influences ΔΨm in mouse cardiac mitochondria, revealing a previously unrecognized role for Hv1 in cardiac mitochondrial physiology. Nevertheless, some limitations of the present study should be considered. In particular, our functional evidence relies primarily on pharmacological inhibition using ClGBI. Although this compound has been extensively employed to investigate Hv1 function [15,16], a recent study has reported that some K^+^ channels and the Nav1.5 channel are sensitive to this drug [17]. However, the concentration of ClGBI used in the present study (10 µM) was lower than the reported inhibitory concentrations of these channels, minimizing off-target effects. The concentration of ClGBI was selected based on the original pharmacological characterization of this compound as an Hv1 inhibitor, in which 10 µM of ClGBI produced a robust inhibition (80–90%) of Hv1 currents [10]. Even assuming that 10 µM ClGBI partially inhibits a mitochondrial K^+^ channel in our experimental preparation, the expected increase in membrane potential would enhance the electrochemical driving force for proton entry into the matrix through Hv1. Therefore, any indirect effect arising from K^+^ channel inhibition would be expected to counteract the matrix alkalinization resulting from Hv1 inhibition. Nevertheless, complementary approaches, including alternative pharmacological tools and genetic models, will be important to further define the molecular identity of the proton conductance described here.

Based on our findings, we propose a working model in which Hv1 resides in the inner mitochondrial membrane in an orientation compatible with proton entry into the mitochondrial matrix, with both the N- and C-termini facing the cytosol. Although its precise mitochondrial localization and membrane topology remain to be experimentally established, such an arrangement would place the channel under the influence of a large membrane potential and transmembrane pH gradient generated during oxidative phosphorylation, both of which favor proton influx into the matrix. At first glance, the large electrochemical gradient across the inner mitochondrial membrane might suggest that Hv1 would behave as a constitutively open proton leak pathway. However, this appears unlikely considering the well-established biophysical properties of Hv1. Channel gating is dynamically regulated by membrane voltage together with the transmembrane pH gradient, which shifts the voltage dependence of activation. In addition, Hv1 activity has been shown to be modulated by several physicochemical factors, including membrane lipids, temperature, and mechanical stimuli [18–20]. Thus, Hv1 could function as a finely tuned proton conductance, dynamically adjusting matrix pH and ΔΨm in response to changing metabolic demands while preserving the proton motive force. Consistent with this concept, we previously demonstrated that ATP directly modulates Hv1 activity [21], suggesting that mitochondrial Hv1 may integrate metabolic cues with proton flux regulation. Additional regulatory mechanisms are likely to exist but remain to be identified.

Overall, our findings provide the first functional evidence for an Hv1-mediated proton conductance in cardiac mitochondria and establish a framework for future studies aimed at defining its molecular identity, regulation, and contribution to cardiac mitochondrial physiology and disease.

## Supplementary Material

Manuscript Supplementary information.docx

## Data Availability

The data that support the findings of this study are available from the corresponding author, VM upon reasonable request.
