# Supplementary material for: Functional evidence of Hv1-mediated proton flux in cardiac mitochondria
Source: Channels (Austin). 2026 Sep 14;20(1):2728231. doi: 10.1080/19336950.2026.2728231 (PMC13577268; doi:10.1080/19336950.2026.2728231)
Supplement: Supplementary file 1 [file KCHL_A_2728231_SM5561.docx]

**Functional evidence of Hv1-mediated proton flux in cardiac mitochondria**

Rayen De Fazio^a^, Clara Ventura^b^, Paulina Finochietto^c^, Ernesto A. Aiello^d^, Carolina Jaquenod De Giusti^e^, Verónica Milesi^f^.

a. Centro de Investigaciones Cardiovasculares ‘Dr. Horacio E. Cingolani’ CCT-La Plata/CONICET, Facultad de Ciencias Médicas, UNLP, La Plata, Argentina, Calle 60 y 120, 1900 La Plata, Argentina. Electronic address [rdefazio@med.unlp.edu.ar](mailto:rdefazio@med.unlp.edu.ar)
b. Instituto de Estudios Inmunológicos y Fisiopatológicos (IIFP), UNLP, CONICET, asociado CIC PBA, Facultad de Ciencias Exactas, La Plata, Argentina. Electronic address: [cventura.conicet@gmail.com](mailto:cventura.conicet@gmail.com)
c. Instituto de Estudios Inmunológicos y Fisiopatológicos (IIFP), UNLP, CONICET, asociado CIC PBA, Facultad de Ciencias Exactas, La Plata, Argentina. Electronic address: [paufinochietto@gmail.com](mailto:paufinochietto@gmail.com)
d. Centro de Investigaciones Cardiovasculares ‘Dr. Horacio E. Cingolani,’ CCT-La Plata/CONICET, Facultad de Ciencias Médicas, UNLP, La Plata, Argentina, Calle 60 y 120, 1900 La Plata, Argentina. Electronic address: [aaiello@med.unlp.edu.ar](mailto:aaiello@med.unlp.edu.ar)
e. Centro de Investigaciones Cardiovasculares ‘Dr. Horacio E. Cingolani’ CCT-La Plata/CONICET, Facultad de Ciencias Médicas, UNLP, La Plata, Argentina, Calle 60 y 120, 1900 La Plata, Argentina. Electronic address: [carolinaj@biol.unlp.edu.ar](mailto:carolinaj@biol.unlp.edu.ar)
f. Instituto de Estudios Inmunológicos y Fisiopatológicos (IIFP), UNLP, CONICET, asociado CIC PBA, Facultad de Ciencias Exactas, La Plata, Argentina. Electronic address: [veronica@biol.unlp.edu.ar](mailto:veronica@biol.unlp.edu.ar) *Corresponding author*

**SUPPLEMENTAL INFORMATION**

**MATERIALS AND METHODS**

***Assessment of mitochondrial purity by immunoblotting.***

Isolated mitochondrial fractions were suspended in RIPA buffer supplemented with protease and phosphatase inhibitors. Equal amounts of protein were separated by SDS-PAGE and transferred to PVDF membranes. After blocking, membranes were incubated overnight at 4°C with anti-VDAC (Invitrogen, PA1-954A, 1:500) and anti-Na⁺/K⁺-ATPase (Santa Cruz Biotechnology, sc-28800, 1:2000) primary antibodies. Following incubation with horseradish peroxidase (HRP)-linked anti-rabbit IgG (Santa Cruz Biotechnology, sc-2317, 1:5000) of anti-mouse IgG (Thermo Fisher, 62-6520, 1:4000) for 1 h at room temperature, protein bands were visualized by chemiluminescence. Mitochondrial purity was assessed by the enrichment of VDAC and the absence of Na⁺/K⁺-ATPase in the mitochondrial fraction.

***Cell culture***

Jurkat T cells were grown in DMEM high-glucose (25 mM) medium supplemented with 10% (v/v) heat-inactivated fetal bovine serum (Internegocios), in 5% CO_2_/95% humidified air at 37 °C at an average density of 10^6^ cells/ml.

***Immunoblot analysis of Hv1 expression in Jurkat cells.***

Jurkat cell lysates were prepared in RIPA buffer supplemented with protease and phosphatase inhibitors and obtained by sonication. Equal amounts of protein were resolved by SDS-PAGE and transferred to PVDF membranes. After blocking, membranes were incubated overnight at 4°C with antibodies against Hv1 (Invitrogen, PA5-24964 1:500) and GAPDH (Ambion, AM4300, 1:1000). After washing, membranes were incubated with horseradish peroxidase (HRP)-linked anti-rabbit IgG (Santa Cruz Biotechnology, sc-2317, 1:5000) of anti-mouse IgG (Thermo Fisher, 62-6520, 1:4000) for 1 h at room temperature. Protein bands were visualized with Chemiluminescent HRP Substrate (Millipore) using a ChemiDoc Image Station (Bio-Rad).
